# Development and validation of a LASSO-selected machine learning prediction pipeline for predicting coronary artery calcification in a health check-up population

**DOI:** 10.3389/fmed.2026.1910240

**Published:** 2026-09-17

**Authors:** Luyu Li, Na Guo, Yuqiang Zuo, Xing Guan, Weinian Gao

**Affiliations:** 1 Department of Cardiac Surgery, The Second Hospital of Hebei Medical University, Shijiazhuang, Hebei, China; 2 Department of Geriatrics, Shijiazhuang Hospital of Traditional Chinese Medicine, Shijiazhuang, Hebei, China; 3 Department of Physical Examination Center, The Second Hospital of Hebei Medical University, Shijiazhuang, Hebei, China; 4 Department of Physical Examination Center, Shijiazhuang People’s Hospital, Shijiazhuang, Hebei, China

**Keywords:** coronary artery calcification, decision curve analysis, health check-up, LASSO, machine learning, SHAP

## Abstract

**Background:**

Coronary artery calcification (CAC) screening in young, asymptomatic populations is clinically valuable but constrained by CT radiation and cost. We developed and calibrated a parsimonious, interpretable machine learning model using routine health check-up variables to identify CAC-positive individuals suitable for early preventive lifestyle counseling.

**Methods:**

A total of 4,465 participants were randomly divided into a training set (*n* = 3,125) and a testing set (*n* = 1,340) using stratified sampling based on CAC status. Candidate predictors included demographic characteristics, clinical history, anthropometric measurements, blood pressure, and laboratory indices. Least absolute shrinkage and selection operator (LASSO) regression was applied in the training set for feature selection, followed by the construction of a class-weighted and probability-calibrated logistic regression model. Model performance was evaluated using the area under the receiver operating characteristic curve (AUC), calibration curves, the Hosmer-Lemeshow test, decision curve analysis (DCA), and SHAP interpretation.

**Results:**

LASSO selected 13 predictors: sex, age, pulse, systolic blood pressure (SBP), diastolic blood pressure (DBP), hypertension (HTN), diabetes mellitus (DM), low-density lipoprotein cholesterol (LDL-C), serum creatinine (Scr), serum uric acid (SUA), non-alcoholic fatty liver disease (NAFLD), smoking, and drinking. The final model achieved an AUC of 0.816 in the training set and 0.771 (95% CI, 0.713−0.824) in the testing set. At the optimal classification threshold, the testing-set sensitivity, specificity, accuracy, PPV, NPV, F1 score, Brier score, and AUPRC were 0.726, 0.689, 0.691, 0.102, 0.981, 0.179, 0.042, and 0.147, respectively. Calibration was acceptable in both cohorts. DCA suggested positive net benefit within a low threshold probability range relevant to preventive counseling and further cardiovascular risk assessment. SHAP analysis identified age, sex, hypertension, pulse, and NAFLD as the most influential predictors.

**Conclusions:**

The LASSO-selected and calibrated logistic regression model demonstrated acceptable discrimination and calibration for CAC positivity in an internally validated health check-up cohort. Its potential use should be interpreted as preliminary risk stratification rather than clinical implementation, and independent multicenter external validation is required before the model can be recommended for routine clinical use.

## Introduction

Cardiovascular disease remains the leading contributor to global morbidity and mortality (1, 2). Coronary artery calcification (CAC) reflects the cumulative burden of coronary atherosclerosis and holds a strong prognostic association with future cardiovascular events across diverse sex and ethnic cohorts (3–6). CAC scoring has been shown to improve cardiovascular risk stratification and is incorporated into contemporary primary prevention guidelines as a risk-refinement tool, particularly when treatment decisions remain uncertain after conventional risk assessment (4, 7, 8). However, universal CAC CT screening is not routinely recommended in low-risk health check-up populations because of radiation exposure, cost, and logistical considerations. Consequently, a parsimonious and interpretable prediction model based on readily available clinical and laboratory indicators is highly desirable to pre-screen individuals with a high probability of CAC positivity, thereby optimizing targeted lifestyle modifications and further cardiovascular risk assessment. While existing cardiovascular risk scores (e.g., Framingham, PCE) often underestimate risk in young asymptomatic cohorts, routine health check-up indices—including blood pressure, lipid profiles, and renal-hepatic metabolic markers—offer an opportunistic window for pre-screening. In the present study, CAC positivity was defined as any detectable coronary calcium (Agatston score > 0 AU), because even minimal CAC may represent early subclinical coronary atherosclerosis and provides incremental risk-stratification value in otherwise low-risk or asymptomatic populations (9, 10).

Traditional regression models provide interpretable associations but frequently suffer from multicollinearity among metabolic and laboratory variables. In particular, lipid indices and insulin-resistance surrogate markers often share mathematical components, while the estimated glomerular filtration rate (eGFR) is derived partly from age, sex, and serum creatinine. Such redundancy can destabilize coefficient estimates in standard regression and lead to overfitting in highly flexible non-linear algorithms. Least absolute shrinkage and selection operator (LASSO) regression addresses this by shrinking redundant predictors toward zero, isolating a parsimonious feature set that offers superior generalization and lower overfitting risk compared with high-capacity tree-based or non-linear machine learning models in sample-constrained clinical datasets (11, 12). To complement this, the SHapley Additive exPlanations (SHAP) framework provides unified, individual-level feature attributions grounded in cooperative game theory (13). Unlike “black-box” non-linear models, SHAP enhances the transparency and clinical trustworthiness of the predictions without sacrificing quantitative accuracy (14). In this study, we followed the TRIPOD guidelines to develop and validate a LASSO-selected prediction model, comparing its predictive performance, calibration, and interpretability against alternative machine learning algorithms (12, 15, 16).

## Methods

### Study population

This retrospective study included participants from a health check-up population with available CAC status and clinical examination data from the Second Hospital of Hebei Medical University, covering the period from January 2025 to December 2025. The study protocol was conducted in accordance with the Declaration of Helsinki and was approved by the Institutional Ethics Committee of the Second Hospital of Hebei Medical University (No.2025-R468). CAC was coded as a binary outcome (0 = negative, 1 = positive). CAC positivity defined by the presence of coronary artery calcification in the CT imaging. Participants were restricted to those aged 35–45 years with available chest CT data. Among 17,353 participants in the merged CT cohort, 5,207 met the age criterion and had available CAC status. Participants with incomplete data for any candidate predictor were excluded before model development, and extreme laboratory values were further removed before construction of the final analytic dataset. The final analytic dataset included 4,465 participants, of whom 206 were CAC positive. The dataset was randomly divided into a training set and a testing set at a 7:3 ratio using stratified sampling according to CAC status. The training set included 3,125 participants with 144 CAC-positive cases, and the testing set included 1,340 participants with 62 CAC-positive cases.

### Candidate predictors and feature selection

Candidate predictors included sex, age, body mass index (BMI), pulse, systolic blood pressure(SBP), diastolic blood pressure(DBP), hypertension(HTN), diabetes mellitus(DM), triglycerides(TG), low-density lipoprotein cholesterol (LDL-C), total cholesterol(TC), high-density lipoprotein cholesterol (HDL-C), fasting plasma glucose(FPG), urea, serum creatinine(Scr), serum uric acid(SUA), non-alcoholic fatty liver disease (NAFLD), smoking, drinking, and eGFR. These variables were selected based on their documented roles in vascular calcification and atherosclerosis (15–17). Continuous variables were standardized during model training. The complete-case approach was used for model development. Participants with missing values in any candidate predictor were excluded before model training; therefore, the final analytic dataset contained no missing values. To evaluate potential selection bias introduced by this approach, missing-data proportions were calculated in the eligible age-restricted CT cohort, and available baseline characteristics were compared between included participants and participants excluded because of incomplete candidate-predictor data. To reduce redundancy and multicollinearity, LASSO logistic regression with 10-fold cross-validation was performed exclusively in the training set. The selected variables were then used for final model construction, model development and validation.

### Model development and validation

A class-weighted logistic regression model with L2 regularization was fitted using the LASSO-selected variables. Class weights were estimated from the distribution of CAC status in the training set only and were applied during model fitting to reduce the influence of class imbalance. To ensure calibrated probability estimates, sigmoid calibration (Platt scaling) was performed within the training set only. Specifically, the calibration model was fitted using cross-validation within the training data, and the held-out testing set was not used for feature selection, class-weight estimation, model fitting, hyperparameter tuning, or calibration-parameter estimation. The testing set was used only for final performance evaluation. Because logistic regression was evaluated alongside SVM, RF, XGBoost, and DT within a supervised prediction framework, the final model is described as a LASSO-selected and calibrated logistic regression model developed within a machine-learning prediction pipeline. Model performance was evaluated in the independent testing set. Discrimination was assessed using the area under the receiver operating characteristic curve (AUC), with bootstrap-derived 95% confidence intervals. Accuracy, sensitivity, specificity, positive predictive value (PPV), negative predictive value (NPV), F1 score, area under the precision-recall curve (AUPRC), and Brier score were also calculated. Calibration was evaluated using calibration curves, the Hosmer-Lemeshow goodness-of-fit test, Brier score, calibration intercept, and calibration slope. Calibration intercept and slope were estimated by fitting a logistic calibration model with CAC status as the dependent variable and the logit-transformed predicted probability as the independent variable. An intercept close to 0 and a slope close to 1 were considered indicative of better calibration. Clinical utility was assessed using decision curve analysis (DCA) across threshold probabilities from 0.00 to 0.20. This low threshold range was selected *a priori* because the prevalence of CAC positivity was low and because the intended clinical action was risk stratification, lifestyle counseling, and consideration of further cardiovascular risk assessment rather than direct pharmacological or invasive decision-making.

### Model interpretation

SHAP analysis was used to quantify the contribution of each selected feature to model prediction. Mean absolute SHAP values were used to rank global feature importance, and SHAP summary plots were used to visualize the direction and magnitude of feature effects.

### Statistical analysis

Continuous variables with normal distribution are presented as mean ± SD, and non-normally distributed variables are presented as median (IQR), and categorical variables are presented as *n* (%). Between-group comparisons were performed using Welch's *t* test for continuous variables and the chi-square or Fisher's exact test for categorical variables. All reported numeric values were rounded to three decimal places. Testing-set AUCs were reported with bootstrap-derived 95% confidence intervals. Pairwise comparisons of testing-set AUCs between the final logistic regression model and alternative machine-learning models were performed using an approximate z test based on the standard errors derived from the bootstrap confidence intervals. The incremental predictive value of TyG, METS-IR, and eGFR was assessed by adding each variable individually and jointly to the final model and comparing testing-set AUC, AUPRC, and Brier score with those of the base final model. Collinearity was assessed using Spearman correlation coefficients and variance inflation factors (VIFs). Analyses were performed using Python (version 3.x) with scikit-learn, scipy, pandas, matplotlib, python-docx, and SHAP.

## Results

### Baseline characteristics

The overall cohort consisted of 4,465 participants. The CAC-positive prevalence was 4.61% in the overall population, 4.61% in the training set, and 4.63% in the testing set. Baseline characteristics of the overall population, training set, and testing set are presented in Table 1. The stratified random split preserved the distribution of CAC status and major baseline variables between the training and testing sets. In the training cohort, participants with CAC positivity were older and harbored a higher burden of cardiometabolic risk factors than their CAC-negative counterparts (Table 2). In the missing-data audit, 5,207 participants aged 35–45 years had available CAC status. Among them, 683 participants were excluded because of incomplete candidate-predictor data, and 59 additional participants were removed because of extreme laboratory values, leaving 4,465 participants in the final analytic dataset. The highest missing-data proportions were observed for fasting plasma glucose (634/5,207, 12.18%), renal biochemical markers including urea, serum creatinine, and serum uric acid (562/5,207, 10.79%), and lipid variables including TG, LDL-C, TC, and HDL-C (546/5,207, 10.49%). Sex, age, BMI, SBP, DBP, hypertension, diabetes mellitus, NAFLD, smoking, and drinking had no missing values. Compared with participants included in the final analytic dataset, those excluded because of incomplete candidate-predictor data had a higher CAC positivity rate (7.03% vs. 4.61%, *P* = 0.010) and differed in several cardiometabolic characteristics, including TG, hypertension, diabetes mellitus, and smoking status (Supplementary Table S1).

**Table 1:** Baseline characteristics of the overall population, training set, and testing set.

Variables	Overall (*n* = 4,465)	Training set (*n* = 3,125)	Testing set (*n* = 1,340)	*P* value
Sex, male	2,227 (49.88)	1,551 (49.63)	676 (50.45)	0.62
Age, years	40.00 (37.00, 43.00)	40.00 (37.00, 43.00)	40.00 (37.00, 43.00)	0.65
BMI, kg/m^2^	24.77 (22.34, 27.36)	24.72 (22.29, 27.43)	24.82 (22.48, 27.29)	0.67
Pulse, bpm	80.00 (73.00, 88.00)	80.00 (73.00, 88.00)	81.00 (74.00, 88.00)	0.75
SBP, mmHg	121.00 (111.00, 132.00)	121.00 (111.00, 133.00)	121.00 (111.00, 132.00)	0.77
DBP, mmHg	77.00 (69.00, 86.00)	77.00 (69.00, 86.00)	76.00 (68.75, 85.00)	0.33
Hypertension	937 (20.98)	645 (20.64)	292 (21.79)	0.39
Diabetes mellitus	152 (3.40)	105 (3.36)	47 (3.51)	0.80
TG, mmol/L	1.20 (0.86, 1.82)	1.20 (0.85, 1.82)	1.20 (0.86, 1.83)	0.90
LDL-C, mmol/L	2.85 (2.39, 3.36)	2.84 (2.39, 3.36)	2.88 (2.40, 3.37)	0.30
TC, mmol/L	4.78 (4.26, 5.37)	4.76 (4.26, 5.35)	4.80 (4.26, 5.41)	0.34
HDL-C, mmol/L	1.37 (1.20, 1.57)	1.38 (1.20, 1.57)	1.37 (1.20, 1.58)	0.70
FPG, mmol/L	4.99 (4.69, 5.34)	4.99 (4.70, 5.33)	5.00 (4.69, 5.36)	0.63
Urea, mmol/L	4.20 (3.59, 4.97)	4.21 (3.59, 4.98)	4.19 (3.59, 4.95)	0.73
Scr, umol/L	72.60 (62.90, 84.10)	72.70 (62.70, 84.10)	72.60 (63.30, 84.50)	0.56
SUA, umol/L	333.00 (271.00, 410.00)	334.00 (272.00, 408.00)	330.00 (270.00, 414.00)	0.88
NAFLD	1,445 (32.36)	1,004 (32.13)	441 (32.91)	0.61
Smoking	883 (19.78)	609 (19.49)	274 (20.45)	0.46
Drinking	1,605 (35.95)	1,121 (35.87)	484 (36.12)	0.88
eGFR, mL/min/1.73m^2^	106.45 (96.09, 113.36)	106.56 (96.18, 113.47)	106.06 (95.91, 112.96)	0.57

**Table 2:** Baseline characteristics of the training set according to CAC status.

Variables	Training overall (*n* = 3,125)	CAC positive (*n* = 144)	CAC negative (*n* = 2,981)	*P* value
Sex, male	1,551 (49.63)	125 (86.81)	1,426 (47.84)	<0.01
Age, years	40.00 (37.00, 43.00)	43.00 (40.00, 44.00)	40.00 (37.00, 42.00)	<0.01
BMI, kg/m^2^	24.72 (22.29, 27.43)	26.87 (24.24, 29.45)	24.63 (22.21, 27.31)	<0.01
Pulse, bpm	80.00 (73.00, 88.00)	79.00 (72.00, 89.00)	80.00 (73.00, 88.00)	0.39
SBP, mmHg	121.00 (111.00, 133.00)	133.50 (120.00, 148.00)	121.00 (111.00, 132.00)	<0.01
DBP, mmHg	77.00 (69.00, 86.00)	88.00 (77.75, 97.00)	77.00 (68.00, 85.00)	<0.01
Hypertension	645 (20.64)	80 (55.56)	565 (18.95)	<0.01
Diabetes mellitus	105 (3.36)	20 (13.89)	85 (2.85)	<0.01
TG, mmol/L	1.20 (0.85, 1.82)	1.69 (1.11, 2.53)	1.18 (0.85, 1.78)	<0.01
LDL-C, mmol/L	2.84 (2.39, 3.36)	3.02 (2.56, 3.55)	2.82 (2.39, 3.35)	<0.01
TC, mmol/L	4.76 (4.26, 5.35)	4.92 (4.45, 5.56)	4.76 (4.24, 5.34)	<0.01
HDL-C, mmol/L	1.38 (1.20, 1.57)	1.27 (1.13, 1.47)	1.38 (1.20, 1.57)	<0.01
FPG, mmol/L	4.99 (4.70, 5.33)	5.25 (4.86, 5.83)	4.98 (4.69, 5.32)	<0.01
Urea, mmol/L	4.21 (3.59, 4.98)	4.54 (3.73, 5.28)	4.19 (3.58, 4.96)	<0.01
Scr, umol/L	72.70 (62.70, 84.10)	83.65 (75.68, 89.92)	72.00 (62.40, 83.50)	<0.01
SUA, umol/L	334.00 (272.00, 408.00)	392.50 (343.75, 459.75)	331.00 (269.00, 405.00)	<0.01
NAFLD	1,004 (32.13)	70 (48.61)	934 (31.33)	<0.01
Smoking	609 (19.49)	58 (40.28)	551 (18.48)	<0.01
Drinking	1,121 (35.87)	96 (66.67)	1,025 (34.38)	<0.01
eGFR, mL/min/1.73m^2^	106.56 (96.18, 113.47)	101.54 (93.55, 109.76)	106.87 (96.40, 113.60)	<0.01

### Feature selection and model performance

LASSO regression selected 13 predictors for final model development: Sex, Age, Pulse, SBP, DBP, HTN, DM, LDL, Scr, SUA, NAFLD, Smoke, and Drink. Table 3 reports model performance in both the training and testing sets. Based on the testing-set AUC, LR showed the best overall discrimination and was selected as the optimal model (Figure 1). Pairwise comparisons of testing-set AUCs showed no statistically significant differences between LR and SVM, RF, XGBoost, or DT (all *P* > 0.05). Therefore, although LR achieved the highest testing-set AUC numerically, its selection as the final model was based on the combined consideration of discrimination, smaller train-test performance gap, calibration, parsimony, and clinical interpretability rather than a statistically significant AUC advantage alone. Incremental-value analysis showed that adding TyG, METS-IR, or eGFR to the final model did not meaningfully improve testing-set performance. Compared with the base final model, the change in testing-set AUC was −0.000 for TyG (*P* = 0.720), −0.003 for METS-IR (*P* = 0.278), and +0.002 for eGFR (*P* = 0.551). Joint addition of TyG, METS-IR, and eGFR also did not improve discrimination (*Δ*AUC = −0.002, *P* = 0.675), with only minimal changes in AUPRC and Brier score. Detailed incremental-value and collinearity results are presented in Supplementary Table S2.

**Table 3:** Performance of difference models.

Model	Dataset	AUC (95% CI)	Accuracy	Specificity	Sensitivity	PPV	NPV	F1 score	Brier score	AUPRC
LR	Training set	0.816 (0.781–0.849)	0.715	0.710	0.806	0.118	0.987	0.207	0.040	0.211
LR	Testing set	0.771 (0.713–0.824)	0.691	0.689	0.726	0.102	0.981	0.179	0.042	0.147
SVM	Training set	0.937 (0.919–0.953)	0.936	0.941	0.833	0.404	0.992	0.544	0.038	0.384
SVM	Testing set	0.757 (0.694–0.815)	0.651	0.646	0.758	0.094	0.982	0.168	0.042	0.147
RF	Training set	0.899 (0.877–0.920)	0.833	0.834	0.826	0.193	0.990	0.314	0.125	0.406
RF	Testing set	0.746 (0.682–0.809)	0.825	0.840	0.532	0.139	0.974	0.220	0.043	0.122
XGBoost	Training set	0.952 (0.941–0.961)	0.815	0.807	0.972	0.196	0.998	0.326	0.111	0.512
XGBoost	Testing set	0.754 (0.689–0.816)	0.700	0.700	0.710	0.103	0.980	0.180	0.042	0.143
DT	Training set	0.846 (0.823–0.868)	0.639	0.624	0.938	0.108	0.995	0.193	0.179	0.170
DT	Testing set	0.746 (0.680–0.810)	0.616	0.612	0.694	0.080	0.976	0.143	0.192	0.141

**Figure 1:**
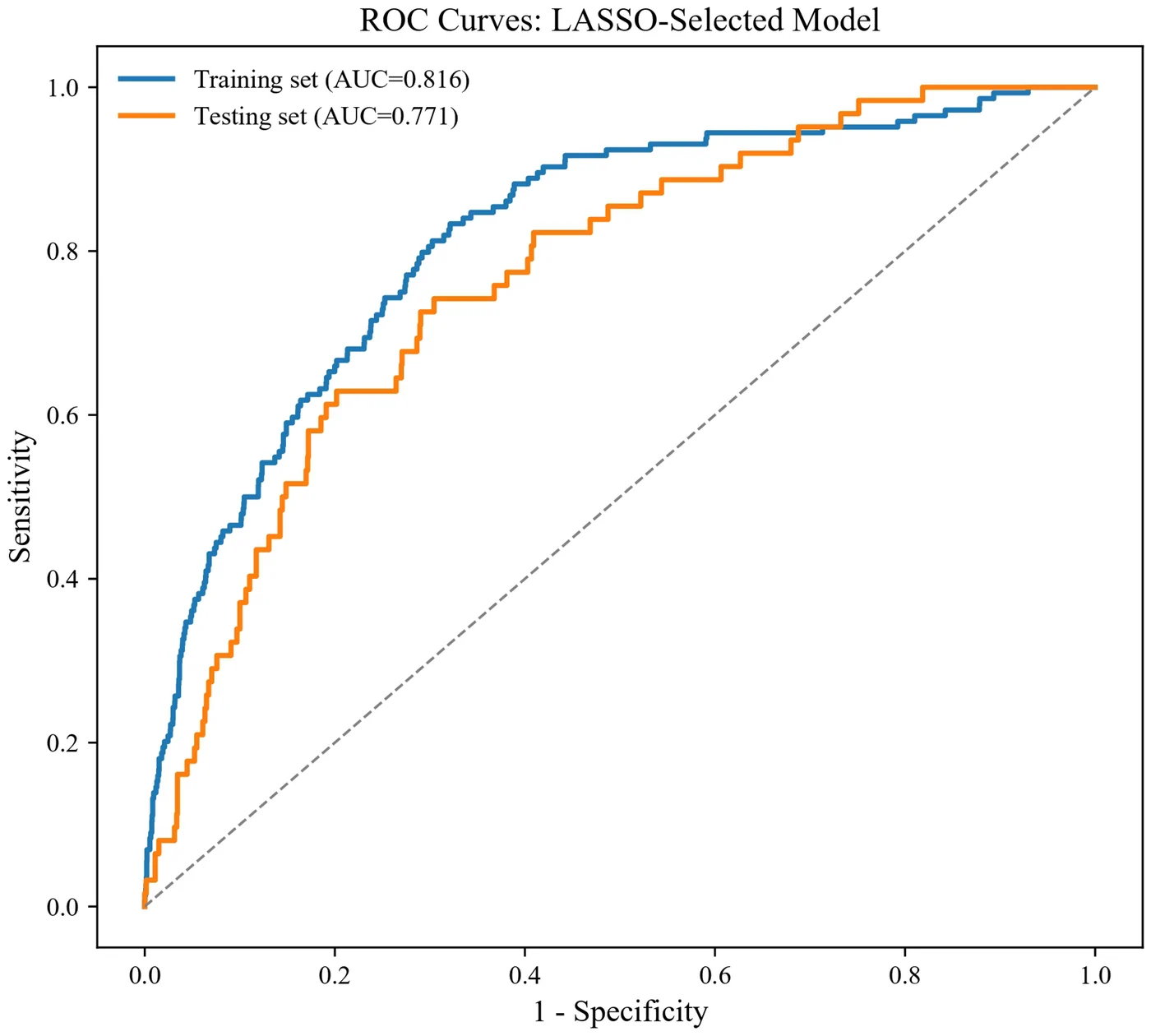
Receiver operating characteristic curves of the LASSO-selected model in the training and testing sets. The model showed acceptable discrimination, with AUC values of 0.816 and 0.771 in the training and testing sets, respectively.

Compared with the more flexible non-linear algorithms, the LASSO-selected logistic regression model showed the most stable generalization performance. The train-test AUC gaps were 0.045 for LR, 0.180 for SVM, 0.153 for RF, 0.198 for XGBoost, and 0.100 for DT. Although SVM, RF, and XGBoost achieved higher apparent AUCs in the training set, their larger decreases in testing performance suggested greater overfitting. LR was therefore selected as the final model because it achieved the highest testing-set AUC, the smallest generalization gap, acceptable calibration, and better clinical interpretability. The modest and statistically non-significant AUC differences indicate that the alternative models did not provide a clinically meaningful improvement in discrimination over the logistic regression model.

### Calibration and clinical utility

The calibration curves showed acceptable agreement between predicted and observed CAC positivity in the low-risk range relevant to health check-up screening (Figure 2). The Hosmer-Lemeshow test indicated no significant lack of fit in either the training set (*χ*^2^ = 10.328, *P* = 0.243) or the testing set (*χ*^2^ = 7.241, *P* = 0.511). Quantitative calibration assessment further showed a calibration intercept of 0.422 and slope of 1.169 in the training set, and a calibration intercept of 0.031 and slope of 1.006 in the testing set. The testing-set calibration intercept was close to 0 and the calibration slope was close to 1, supporting acceptable calibration of predicted probabilities. DCA showed that the model provided positive net benefit across low threshold probabilities (Figure 3). Given the low prevalence of CAC positivity in this health check-up cohort, this finding should be interpreted as supporting risk stratification and low-risk preventive counseling rather than direct treatment or invasive testing decisions.

**Figure 2:**
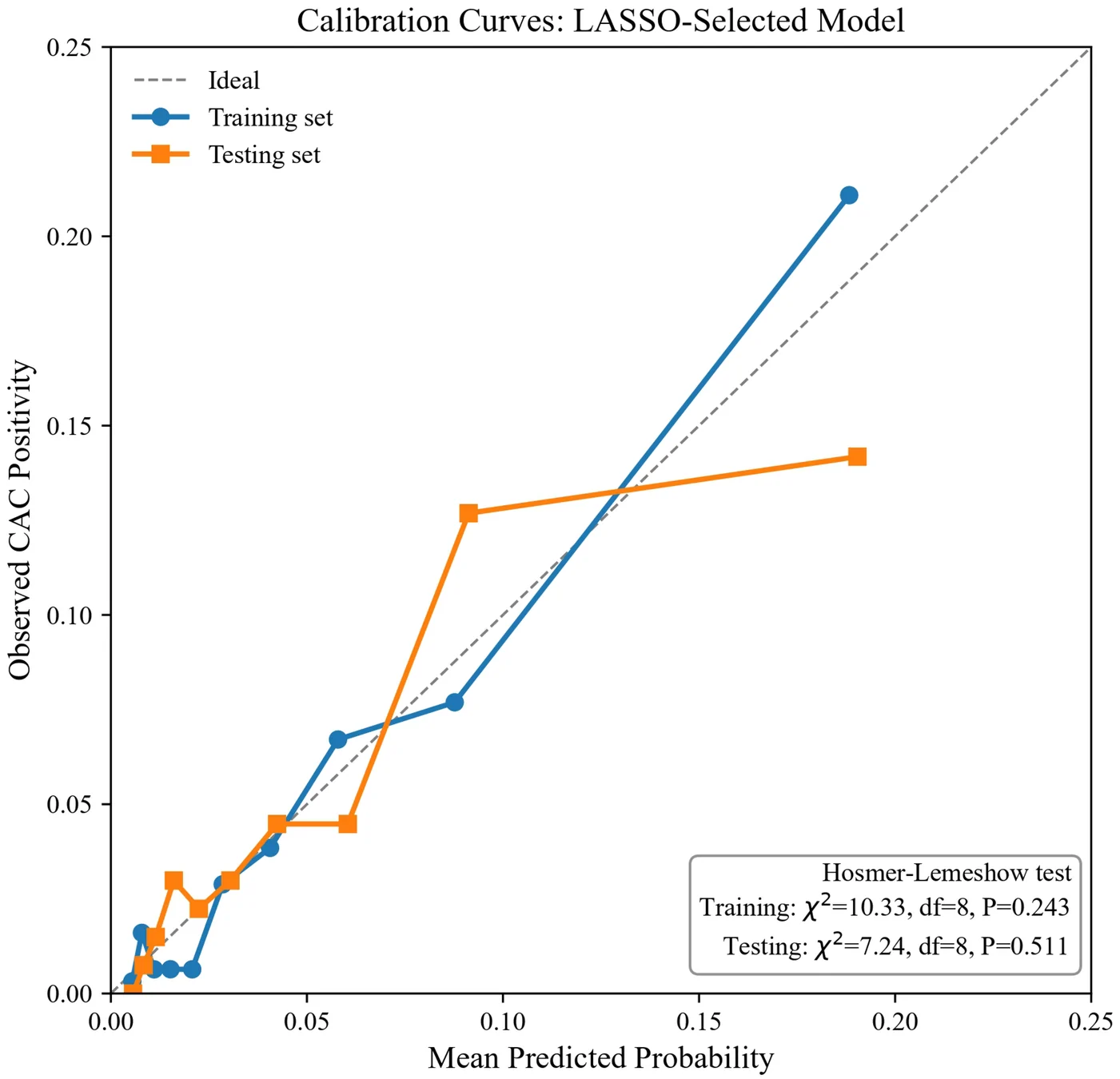
Calibration curves of the LASSO-selected logistic model in the training and testing sets. The grey dashed line represents ideal perfect calibration (where predicted probabilities match observed CAC positivity rates). The inserted box shows the Hosmer-Lemeshow goodness-of-fit test results, confirming acceptable calibration in both cohorts (*P* > 0.05). Calibration intercept and slope were 0.422 and 1.169 in the training set, and 0.031 and 1.006 in the testing set, respectively.

**Figure 3:**
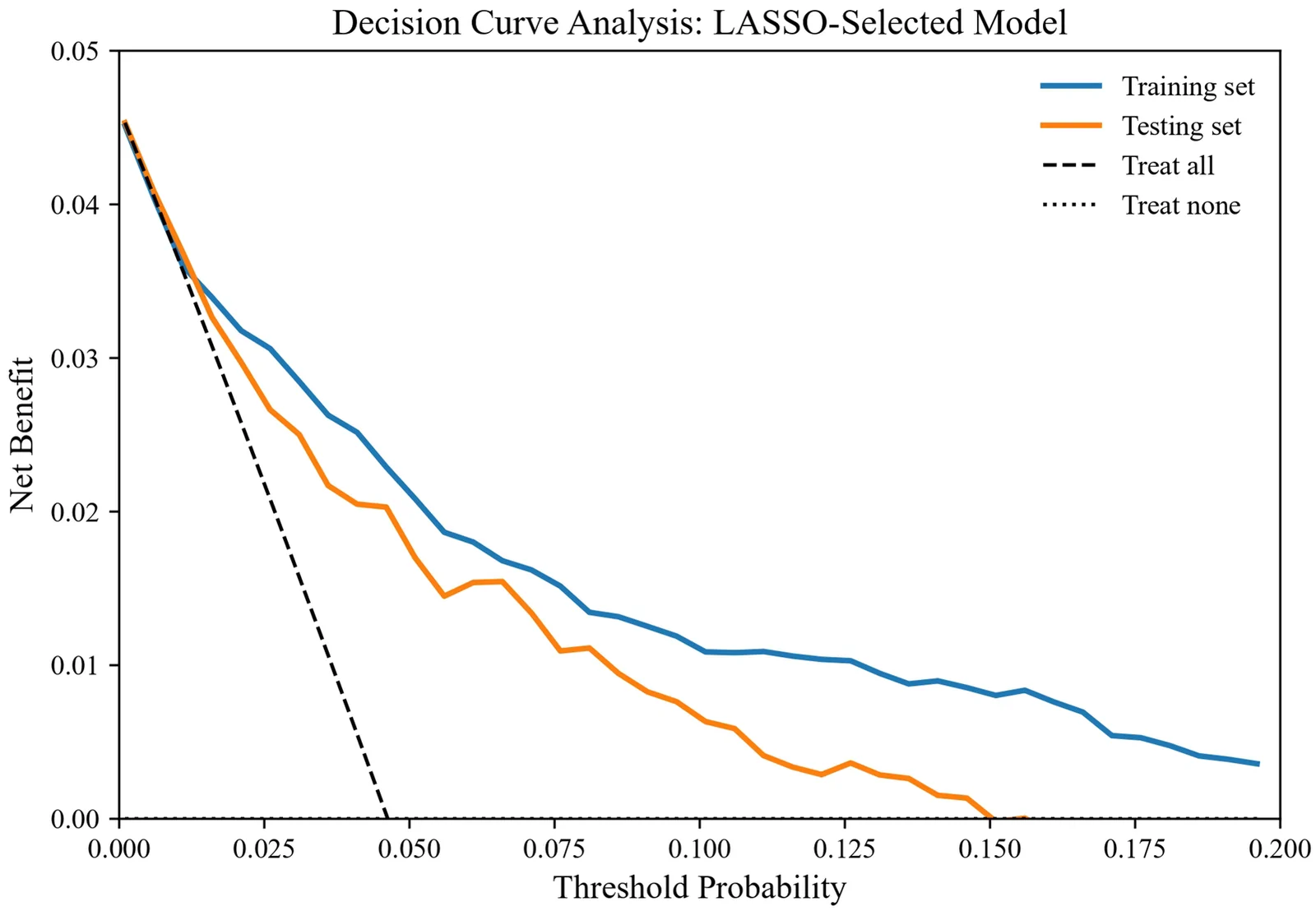
Decision curve analysis (DCA) for the LASSO-selected model. The *x*-axis indicates the threshold probability, and the *y*-axis indicates net benefit. The treat-all line assumes that all participants are considered CAC-positive, whereas the treat-none line assumes that no participant is considered CAC-positive. The model showed positive net benefit within the clinically relevant low-threshold probability range, supporting its use for preliminary risk stratification rather than diagnostic decision-making.

### SHAP-based model interpretation

SHAP analysis was used to explain the final model. The top five variables ranked by mean absolute SHAP values were age, sex, HTN, pulse, and NAFLD (Figures 4, 5). These rankings should be interpreted as model-attribution results rather than causal effects. Because several predictors were clinically or mathematically correlated, individual SHAP contributions may be influenced by shared information among related variables. Therefore, the SHAP results indicate which variables contributed most strongly to the fitted model output, but they do not establish independent biological or causal effects.

**Figure 4:**
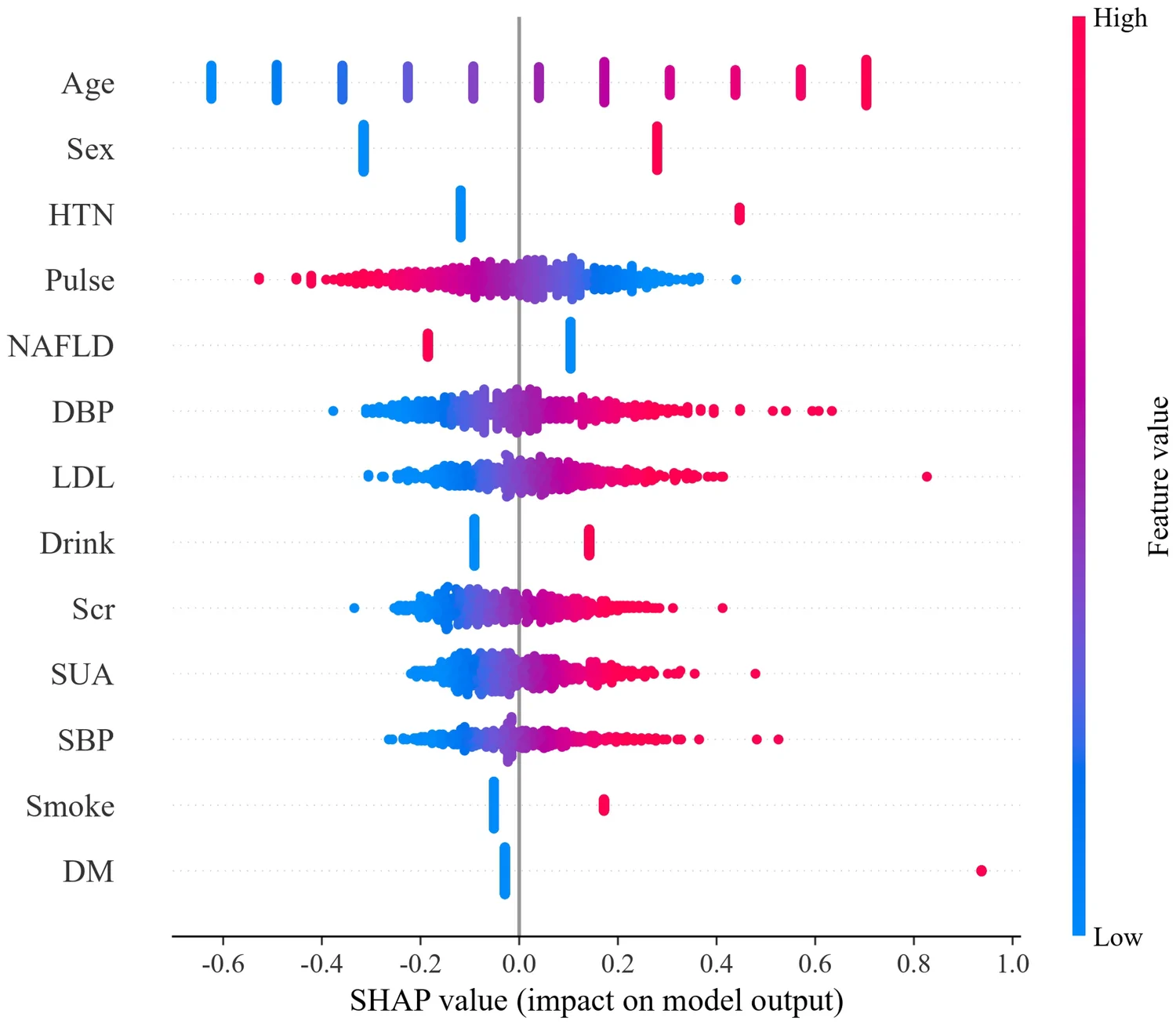
SHAP summary plot for the LASSO-selected model. Each dot represents one participant. The color scale indicates the feature value, with red indicating higher values and blue indicating lower values. The horizontal axis indicates the magnitude and direction of each feature's contribution to the model output. Positive SHAP values indicate a higher model output toward CAC positivity, but do not imply causal effects. Individual SHAP contributions may also be influenced by correlations or shared information among related predictors.

**Figure 5:**
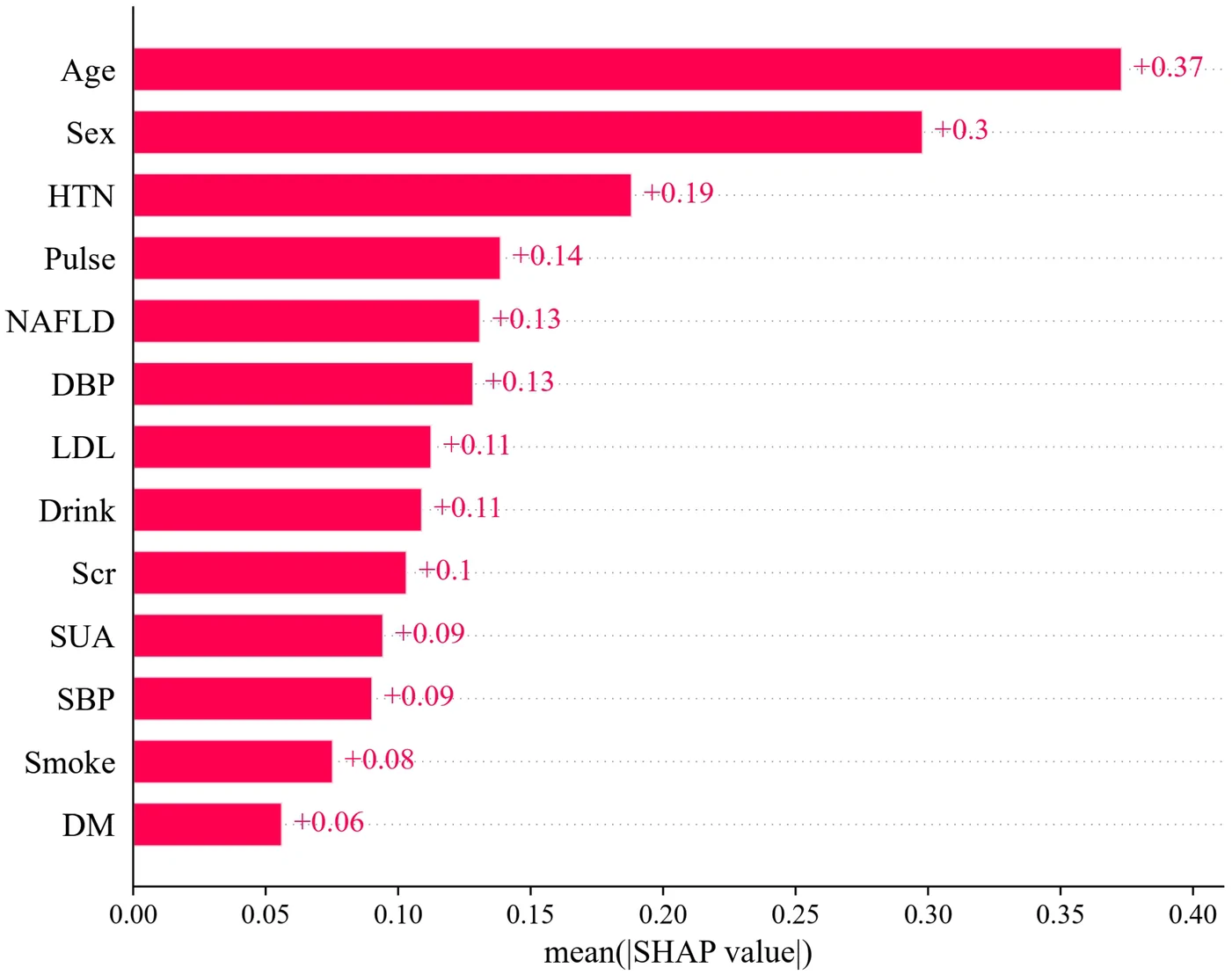
Global SHAP feature importance of the LASSO-selected model ranked by mean absolute SHAP value.

## Discussion

In this health check-up population, we developed and internally validated a LASSO-selected machine learning model for predicting CAC positivity. The final model showed acceptable discrimination in the independent testing set and acceptable calibration, while DCA suggested positive net benefit within low threshold probabilities. These findings support the potential use of routine health check-up variables for preliminary CAC risk stratification and for identifying individuals who may benefit from early lifestyle modification or further cardiovascular risk assessment, rather than for directly guiding pharmacological treatment or invasive testing. Because of the observational and retrospective design, the present study demonstrates predictive associations and model-attribution patterns rather than causal mechanisms. The mechanistic explanations discussed below should therefore be interpreted as biologically plausible hypotheses supported by prior literature, not as mechanisms directly proven by the present data.

From a pathophysiological perspective, prior studies suggest that CAC is not merely a passive mineral deposition process but an actively regulated manifestation of atherosclerotic plaque biology. Vascular smooth muscle cells (VSMCs) undergo osteogenic transdifferentiation under the influence of oxidative stress, inflammatory cytokines, disordered mineral metabolism, lipoprotein retention, and cellular senescence (17–20). Microcalcification typically arises within inflamed plaques and apoptotic cell-rich regions, whereas more extensive calcification reflects chronic plaque remodeling and cumulative exposure to cardiometabolic injury. This prior evidence provides biological plausibility for why a CAC prediction model may capture integrated information from age, blood pressure, lipid metabolism, renal-metabolic status, systemic inflammation, and metabolic comorbidities rather than a single isolated biomarker.

Age had the highest global feature attribution in the SHAP analysis, indicating that it contributed most strongly to model predictions in this dataset. This finding is biologically plausible and is consistent with prior evidence that CAC reflects the cumulative burden of vascular injury, arterial stiffening, cellular senescence, and osteogenic signaling within the vascular wall (4, 17, 21). Sex was also highly ranked, which may reflect well-established differences in CAC distribution and cardiovascular risk trajectories between men and women (4, 5). These differences may relate to hormonal status, fat distribution, vascular remodeling, and differential exposure to traditional risk factors. In a health check-up cohort with relatively low CAC prevalence, age and sex therefore provided foundational risk information that dominated the prediction signal. Importantly, SHAP values quantify feature contributions to model output and should be interpreted as explanations of the fitted prediction model rather than evidence of causal mechanisms. Because age, sex, blood pressure variables, renal-metabolic markers, and lifestyle factors may be interrelated, SHAP attributions for individual variables may also reflect shared predictive information rather than isolated independent effects. In our cohort, sex was one of the most influential SHAP features, and men accounted for a substantially higher proportion of CAC-positive cases. This is consistent with prior Asian evidence indicating sex-related differences in zero-CAC prevalence and progression, with men showing a higher likelihood of subclinical CAC progression than women (22, 23). Accordingly, sex-specific calibration and threshold validation may be warranted before uniform application of the model in men and women.

Hypertension and blood pressure-related variables were important contributors to the model. Hemodynamic load has been reported to be associated with endothelial injury, medial remodeling, arterial stiffness, and cyclic mechanical strain on VSMCs, processes that may contribute to atherosclerotic plaque development and calcific remodeling (24, 25). Pulse and diastolic blood pressure may reflect autonomic tone, vascular stiffness, peripheral resistance, and cardiorespiratory fitness. Their selection by LASSO suggests that blood pressure-related variables provided predictive information beyond binary hypertension status in this dataset. These findings are consistent with longitudinal data showing that blood pressure and related vascular stiffness phenotypes are associated with CAC development and progression (24, 25).

LDL-C remained in the LASSO-selected feature set, a finding consistent with the established role of atherogenic lipoproteins in coronary plaque formation. LDL particles infiltrate the arterial intima, undergo oxidative modification, and promote macrophage foam-cell formation, necrotic core development, and local inflammatory activation. Over time, lipid-rich plaques evolve toward fibrotic and calcified phenotypes. Although lipid variables are often correlated with derived insulin-resistance indices, LDL-C represents a direct atherogenic exposure and is a key therapeutic target in cardiovascular prevention guidelines (7, 8). Its retention in the model is therefore pathophysiologically plausible.

Renal-metabolic markers, including serum creatinine and serum uric acid, also contributed to prediction. Prior studies suggest that even mild renal dysfunction may be associated with phosphate retention, oxidative stress, endothelial dysfunction, chronic inflammation, and dysregulated mineral metabolism, which could plausibly contribute to vascular calcification (26–28). Uric acid has also been linked to oxidative stress, inflammasome activation, endothelial nitric oxide impairment, and renal-metabolic clustering, although these pathways were not directly assessed in the present study (29–31). The selection of these variables suggests that subclinical renal-metabolic stress may help identify individuals with higher CAC risk even in a general health check-up population.

NAFLD was among the important predictors, consistent with the concept that fatty liver disease is a systemic metabolic disorder rather than a liver-limited condition. NAFLD is associated with insulin resistance, visceral adiposity, atherogenic dyslipidemia, low-grade inflammation, oxidative stress, and altered adipokine signaling, all of which may contribute to endothelial dysfunction and atherosclerosis according to prior literature (32–34). Prior studies and meta-analyses have reported associations between NAFLD and CAC (32, 35, 36). In the present model, NAFLD may have functioned as an integrative predictive marker of hepatic-metabolic dysfunction, potentially capturing risk information related to insulin resistance and systemic metabolic inflammation.

An important finding was that LASSO did not retain TyG, METS-IR, or eGFR in the final parsimonious model, despite prior evidence linking these indices to CAC or coronary artery disease. This likely reflects overlapping predictive information rather than biological irrelevance. Consistent with this interpretation, these derived indices were highly correlated with their component or related predictors, and adding them to the final model did not provide meaningful incremental discrimination or calibration. Therefore, LASSO may have preferentially retained more direct or stable predictors, such as age, sex, Scr, lipid variables, and cardiometabolic comorbidities, while excluding derived indices that contributed limited additional information in this dataset.

A notable observation was the performance disparity between complex machine learning algorithms and the logistic model. Although SVM, RF, and XGBoost achieved higher training-set AUCs, their testing-set performance decreased more substantially than that of LR. This pattern is consistent with the bias-variance trade-off: high-capacity models may fit training-set patterns that do not generalize well, especially in tabular clinical data with a low event rate. In contrast, the LASSO-selected and L2-regularized logistic model provided a more parsimonious structure, smaller train-test AUC gap, acceptable calibration, and clearer clinical interpretability.

We acknowledge that the final selected model was a penalized and calibrated logistic regression model rather than a high-complexity machine-learning algorithm. In this study, the term “machine learning” refers to the overall supervised prediction pipeline, which included LASSO-based feature selection, comparison with multiple learning algorithms, class-weighted model fitting, probability calibration, decision-curve evaluation, and SHAP-based model interpretation. The final use of logistic regression was therefore intentional: among the evaluated algorithms, it provided the most favorable balance of discrimination, calibration, generalization stability, parsimony, and clinical interpretability.

Importantly, a CAC score of zero does not fully exclude vulnerable non-calcified plaque or obstructive CAD in selected patients. Prior Asian studies have shown that individuals with baseline zero CAC may later develop subclinical CAC progression, and some individuals with zero CAC may still harbor obstructive CAD ≥50% on CCTA; recent LASSO-based prediction models have also been proposed to identify this high-risk subgroup using routine clinical variables (37). Therefore, the present model should be interpreted as a screening tool for initial risk stratification rather than a rule-out test for coronary disease in individuals with persistent symptoms or a high clinical suspicion. In this low-prevalence screening context, CAC positivity was present in only 4.61% of participants, which substantially constrained the PPV of the model. In the testing set, the PPV of the final LR model was 0.102, indicating that many individuals classified as high risk would not have CAC positivity on CT. This low PPV limits the operational utility of the model as a standalone diagnostic or mass-screening decision tool. Conversely, the high NPV of 0.981 suggests that the model may be more useful for identifying individuals unlikely to have CAC positivity. Therefore, the model should be interpreted primarily as a preliminary risk-stratification tool to guide preventive counseling and consideration of further cardiovascular risk assessment, rather than as a tool for confirming CAC positivity or directly guiding invasive or pharmacological decisions. Future sensitivity analyses using oversampling strategies or cost-sensitive classification may further evaluate whether performance and net benefit can be improved under class imbalance.

This study has several limitations. First, the model was developed and internally validated using single-center data with a train-test split. The absence of external validation limits the generalizability and transportability of the model across different populations, scanners, CT protocols, and health check-up settings. Therefore, the present model should be considered an internally validated preliminary risk-stratification tool rather than a model ready for routine clinical implementation. Independent multicenter validation is required to confirm its discrimination, calibration, clinical utility, and decision thresholds, and recalibration may be necessary before application in local populations. Second, although the final analytic dataset contained no missing values, this was achieved by excluding participants with incomplete candidate-predictor data and extreme laboratory values. Participants excluded because of incomplete candidate-predictor data had a higher CAC positivity rate than those included in the final analytic dataset. This finding is clinically plausible because individuals with underlying comorbidities or abnormal biochemical profiles may have a higher burden of subclinical coronary atherosclerosis. Nevertheless, this pattern suggests that missingness was unlikely to be completely random, and residual selection bias related to complete-case analysis cannot be fully excluded. Third, the retrospective observational design limits causal inference. The mechanistic explanations discussed in this study were based on biological plausibility and prior literature rather than direct mechanistic measurements. Therefore, these explanations should be considered hypothesis-generating and should not be interpreted as causal mechanisms demonstrated by the present data. Fourth, CAC was analyzed as a binary outcome. Future studies may evaluate ordinal or continuous CAC scores to capture disease burden more granularly. Fifth, the event rate was low, which limited the PPV and may affect model transportability. Sixth, LASSO improves parsimony but may exclude biologically meaningful variables when their information overlaps with other predictors. Finally, SHAP values explain model behavior rather than causal effects. Because correlated predictors may share overlapping information, individual SHAP contributions should not be interpreted as independent biological effects, and mechanistic interpretation should therefore be made cautiously.

## Conclusion

A LASSO-selected and calibrated logistic regression model developed within a machine-learning prediction pipeline showed acceptable discrimination, calibration, and positive net benefit within low threshold probabilities for predicting CAC positivity. The model may help identify individuals who could benefit from lifestyle modification and further cardiovascular risk assessment, while its utility should be interpreted primarily in the context of risk stratification. Independent multicenter external validation is required before routine clinical implementation can be recommended.

## Data Availability

The raw data supporting the conclusions of this article will be made available by the authors, without undue reservation.
