# Supplementary material for: Development and validation of a LASSO-selected machine learning prediction pipeline for predicting coronary artery calcification in a health check-up population
Source: Front Med (Lausanne). 2026 Sep 17;13:1910240. doi: 10.3389/fmed.2026.1910240 (PMC13626897; doi:10.3389/fmed.2026.1910240)
Supplement: Supplementary file 1 [file Datasheet1.pdf]

Supplementary Table S1. Missing-data proportions and comparison of included participants with participants excluded because of incomplete candidate-predictor data.

Panel A. Cohort-selection flow

Stage	N	CAC positive, n	CAC positive, %
Source merged CT cohort	17,353	3,696	21.30
Age 35-45 years with CAC status	5,207	263	5.05
Excluded because of incomplete candidate-predictor data	683	48	7.03
Complete candidate-predictor data in age-restricted cohort	4,524	215	4.75
Final analytic dataset after removal of extreme values	4,465	206	4.61

Panel B. Missing-data proportions in the age-restricted CT cohort, n=5,207

Variable	Missing, n	Missing, %
Sex	0	0.00
Age	0	0.00
BMI	0	0.00
Pulse	3	0.06
SBP	0	0.00
DBP	0	0.00
Hypertension	0	0.00
Diabetes mellitus	0	0.00
TG	546	10.49
LDL-C	546	10.49
TC	546	10.49
HDL-C	546	10.49
FPG	634	12.18
Urea	562	10.79
Scr	562	10.79
SUA	562	10.79
NAFLD	0	0.00
Smoking	0	0.00
Drinking	0	0.00

Panel C. Comparison between final included participants and participants excluded because of incomplete candidate-predictor data

Variable	Included participants, n=4,465	Excluded because of missing data, n=683	P value
Age, years	40.00 (37.00, 43.00)	40.00 (38.00, 43.00)	<0.001
BMI, kg/m?	24.77 (22.34, 27.36)	24.62 (22.19, 27.85)	0.699
Pulse, bpm	80.00 (73.00, 88.00)	80.00 (73.00, 88.00)	0.536
SBP, mmHg	121.00 (111.00, 132.00)	121.00 (110.00, 133.00)	0.960
DBP, mmHg	77.00 (69.00, 86.00)	77.00 (69.00, 87.00)	0.835
TG, mmol/L	1.20 (0.86, 1.82)	1.43 (0.89, 2.09)	0.021
LDL-C, mmol/L	2.85 (2.39, 3.36)	2.90 (2.46, 3.48)	0.289
TC, mmol/L	4.78 (4.26, 5.37)	4.84 (4.39, 5.49)	0.245
HDL-C, mmol/L	1.37 (1.20, 1.57)	1.35 (1.19, 1.56)	0.809
FPG, mmol/L	4.99 (4.69, 5.34)	5.14 (4.77, 5.37)	0.239
Urea, mmol/L	4.20 (3.59, 4.97)	4.19 (3.70, 4.85)	0.941
Scr, μmol/L	72.60 (62.90, 84.10)	70.40 (62.20, 83.50)	0.452
SUA, μmol/L	333.00 (271.00, 410.00)	325.00 (264.00, 406.00)	0.328
Sex, male	2,227 (49.88)	329 (48.17)	0.411
Hypertension	937 (20.99)	54 (7.91)	<0.001
Diabetes mellitus	152 (3.40)	9 (1.32)	0.002
NAFLD	1,445 (32.36)	226 (33.09)	0.726
Smoking	883 (19.78)	163 (23.87)	0.014
Drinking	1,605 (35.95)	253 (37.04)	0.578
CAC positivity	206 (4.61)	48 (7.03)	0.010

Note. Continuous variables are presented as median (interquartile range), and categorical variables are presented as n (%). CAC, coronary artery calcification; BMI, body mass index; SBP, systolic blood pressure; DBP, diastolic blood pressure; TG, triglycerides; LDL-C, low-density lipoprotein cholesterol; TC, total cholesterol; HDL-C, high-density lipoprotein cholesterol; FPG, fasting plasma glucose; Scr, serum creatinine; SUA, serum uric acid; NAFLD, non-alcoholic fatty liver disease.
