# Supplementary material for: Development and validation of a LASSO-selected machine learning prediction pipeline for predicting coronary artery calcification in a health check-up population
Source: Front Med (Lausanne). 2026 Sep 17;13:1910240. doi: 10.3389/fmed.2026.1910240 (PMC13626897; doi:10.3389/fmed.2026.1910240)
Supplement: Supplementary file 2 [file Datasheet2.pdf]

Supplementary Table S2. Incremental predictive value and collinearity assessment of TyG, METS-IR, and eGFR

Panel A. Incremental predictive performance after adding derived indices to the base final model

Model	Features added	Testing AUC	Testing AUPRC	Testing Brier	△AUC vs base	△AUC 95% CI	P value
Base final model	nan	0.771	0.196	0.0409	Reference	-	-
Base + TyG	TyG	0.771	0.191	0.0409	-0.000	-0.002 to 0.001	0.720
Base + METS-IR	METS-IR	0.768	0.202	0.0409	-0.003	-0.009 to 0.003	0.278
Base + eGFR	eGFR	0.773	0.202	0.0408	+0.002	-0.004 to 0.008	0.551
Base + TyG + METS-IR + eGFR	TyG, METS-IR, eGFR	0.769	0.205	0.0408	-0.002	-0.013 to 0.008	0.675

Panel B. Collinearity and correlation assessment

Variable pair	Spearman ?	P value	VIF of variable 1	VIF of variable 2
TyG vs TG	0.980	0.000e+0	2.39	-
TyG vs FPG	0.416	1.093e-186	2.39	-
METS-IR vs BMI	0.940	0.000e+0	2.69	-
METS-IR vs TG	0.672	0.000e+0	2.69	-
METS-IR vs HDL	-0.625	0.000e+0	2.69	-
METS-IR vs FPG	0.360	2.197e-136	2.69	-
eGFR vs Age	-0.258	5.740e-69	20.21	1.67
eGFR vs Sex	-0.179	1.276e-33	20.21	18.90
eGFR vs Scr	-0.740	0.000e+0	20.21	40.55

Note. The base final model included Sex, Age, Pulse, SBP, DBP, HTN, DM, LDL, Scr, SUA, NAFLD, Smoke, and Drink. Higher VIF values indicate greater collinearity. TyG, triglyceride-glucose index; METS-IR, metabolic score for insulin resistance; eGFR, estimated glomerular filtration rate; AUC, area under the receiver operating characteristic curve; AUPRC, area under the precision-recall curve; VIF, variance inflation factor.
